# Mitochondrial genome of a brachypterous species in Meconematinae: *Acosmetura nigrogeniculata* and its phylogenetic implication

**DOI:** 10.1080/23802359.2019.1622468

**Published:** 2019-07-10

**Authors:** Ning Han, Hao Yuan, Jing Wang, Yafu Zhou, Shaoli Mao

**Affiliations:** a Shaanxi Key Laboratory for Animal Conservation, Shaanxi Institute of Zoology, Xi’an, China;; b College of Life Sciences, Shaanxi Normal University, Xi’an, China;; c Xi’an Botanical Garden of Shaanxi Province, Institute of Botany of Shaanxi Province, Xi’an, China;; d Shaanxi Engineering Research Centre for Conservation and Utilization of Botanical Resources, Xi’an, China

**Keywords:** *Acosmetura nigrogeniculata*, mitochondrial genome, next-generation sequencing, phylogeny

## Abstract

*Acosmetura nigrogeniculata* (Liu and Wang, 1998) is a brachypterous species in Meconematinae, which is only distributed in China. In this study, the complete mitochondrial genome of *A. nigrogeniculata* was determined and annotated. The 16,271 bp circular genome contained 13 protein-coding genes, 22 transfer RNA genes, two ribosomal RNA genes, and one control region. The overall base composition was 36.4% A, 34.8% T, 18.4% C, and 10.5% G, exhibiting obvious anti-G and AT bias (71.2%). The general genomic characters including nucleotides composition, gene arrangement, and codon usage were similar to those of other Meconematinae species. Phylogenetic analysis of all nine Meconematinae species indicated that the newly sequenced species were clustered closely with the brachypterous species *Pseudosmetura snjiensis*.

Meconematinae is a diverse subfamily in Tettigoniidae (Cigliano et al. 2019). To date, the mitochondrial genomes of six macropterous species and only one brachypterous species in Meconematinae have been determined (Yang et al. 2012; Liu 2017; Zhou et al. 2017; Mao et al. 2018a, 2018b). The specimen of *A. nigrogeniculata* was collected at Neixiang in Henan province (33°29′N, 111°58′E), China in August 2009 and deposited in Institute of Botany of Shaanxi Province. In this study, the complete mitochondrion genome of *A. nigrogeniculata* was sequenced using the next-generation sequencing technology. The whole sequence was annotated using the software Geneious v 11.1.5, and tRNA genes were predicted using online software MITOS (Bernt et al. 2013).

The complete mitogenome of *A. nigrogeniculata* is 16,271 bp in length and has been deposited in GenBank (accession no. MK801775). It consists of 13 protein-coding genes (PCGs), 22 tRNA genes, two rRNA genes, and one control region, and its structure and arrangement are identical with hypothesized ancestral insect mitogenome (Boore 1999). The *A. nigrogeniculata* mitochondrial genes are separated by a total of 121 bp of intergenic spacer sequences, which are spread over 10 regions and range in size from 1 to 45 bp. There are 13 overlaps with all of 48 bp and the longest overlaps (8 bp) locate between *tRNA^Trp^*-*tRNA^Cys^* and *tRNA^Tyr^*-*COI.* The overall base composition of the whole mitochondrial genome is 36.4% A, 34.8% T, 18.4% C, and 10.5% G, exhibiting obvious AT bias (71.2%). The initiation codons of all PCGs are typical ATN (COII, ATP6, COIII, ND4, ND4L, and Cytb with ATG; ND2, COI, ATP8, ND3, ND5 with ATT; ND6 and ND1 with ATA). Seven genes (ND2, COII, ATP8, ATP6, ND3, ND4L, and ND6) use TAA as the termination codons, and two genes (Cytb, ND1) are stopped with TAG. COI, COIII, ND5, and ND4 genes have an incomplete stop codon T––. The relative synonymous codon frequencies analysis shows that the three codon families (Leu (UUR), Ile and Phe) are the most frequently used, totally accounting for about 29% of all the PCG codons. All tRNA genes could be folded into the typical cloverleaf secondary structures except for tRNA^Ser(AGN)^. The lrRNA and srRNA are 1314 bp and 786 bp in length, respectively. They are located between tRNA^Phe^ and tRNA^Leu (UUR)^, being separated by tRNA^Val^. The control region is 1408 bp in length and locates between srRNA and tRNA^Ile^ and is composed of 61.8% A and T nucleotides.

Phylogenetic analysis was performed using MrBayes 3.1.2 (Ronquist and Huelsenbeck 2003) under the partitioned models chosen by PartitionFinder (Lanfear et al. 2017) based on the datasets of 13 PCGs and two rRNAs genes. Bayesian analysis result showed that relationships among genera in Meconematinae were similar to the maximum likelihood analysis result with site-homogeneous model (Mao et al. 2018a, 2018b). The newly sequenced species were clustered with another brachypterous species *Pseudocosmetura anjiensis* with a high bootstrap value (PP = 1), and sister to other four macropterous species (Figure 1).

**Figure 1.**
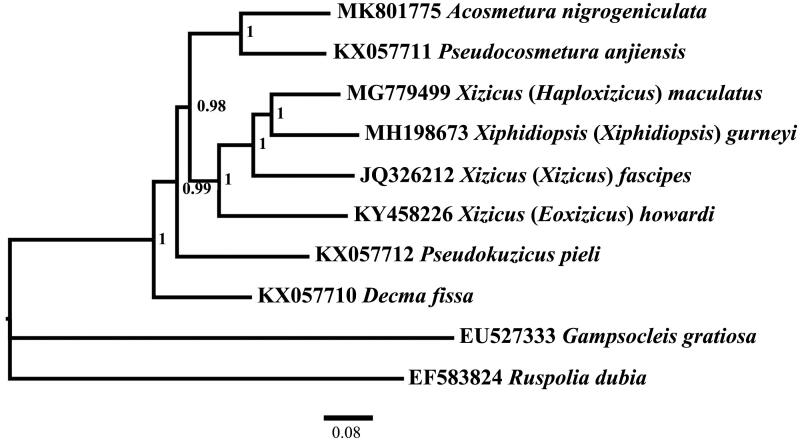
Phylogenetic reconstruction of Meconematinae using mitochondrial PCGs and rRNA concatenated dataset.
